# Model-Based Feasibility Assessment of Membrane Biofilm Reactor to Achieve Simultaneous Ammonium, Dissolved Methane, and Sulfide Removal from Anaerobic Digestion Liquor

**DOI:** 10.1038/srep25114

**Published:** 2016-04-26

**Authors:** Xueming Chen, Yiwen Liu, Lai Peng, Zhiguo Yuan, Bing-Jie Ni

**Affiliations:** 1 Advanced Water Management Centre, The University of Queensland, St. Lucia, Brisbane, QLD 4072, Australia

## Abstract

In this study, the membrane biofilm reactor (MBfR) is proposed to achieve simultaneous removal of ammonium, dissolved methane, and sulfide from main-stream and side-stream anaerobic digestion liquors. To avoid dissolved methane stripping, oxygen is introduced through gas-permeable membranes, which also from the substratum for the growth of a biofilm likely comprising ammonium oxidizing bacteria (AOB), anaerobic ammonium oxidation (Anammox) bacteria, denitrifying anaerobic methane oxidation (DAMO) microorganisms, aerobic methane oxidizing bacteria (MOB), and sulfur oxidizing bacteria (SOB). A mathematical model is developed and applied to assess the feasibility of such a system and the associated microbial community structure under different operational conditions. The simulation studies demonstrate the feasibility of achieving high-level (>97.0%), simultaneous removal of ammonium, dissolved methane, and sulfide in the MBfRs from both main-stream and side-stream anaerobic digestion liquors through adjusting the influent surface loading (or hydraulic retention time (HRT)) and the oxygen surface loading. The optimal HRT was found to be inversely proportional to the corresponding oxygen surface loading. Under the optimal operational conditions, AOB, DAMO bacteria, MOB, and SOB dominate the biofilm of the main-stream MBfR, while AOB, Anammox bacteria, DAMO bacteria, and SOB coexist in the side-stream MBfR to remove ammonium, dissolved methane, and sulfide simultaneously.

Anaerobic technologies are getting increased attention in wastewater treatment due to their low energy demands and energy recovery potential from wastewater. Aerobic-based domestic wastewater treatment in combination with side-stream anaerobic sludge digestion has been widely implemented in wastewater treatment plants (WWTPs) to achieve energy recovery in the form of methane whilst fulfilling the purpose of nutrient removal from wastewater. Direct main-stream anaerobic digestion of domestic wastewater^1^,^2^ is gaining more attention owing to its enhanced energy recovery, which makes it possible for WWTPs to function in an energy-neutral or even energy-generating manner. In order to meet regulatory discharge standards while promoting energy conservation, low-energy demanding downstream processes are required to treat main-stream and side-stream anaerobic digestion liquors. Ammonium represents the major constituent in the anaerobic digestion liquor. The ammonium concentration in the main-stream anaerobic digestion liquor ranges between 9 and 67 mg N L^−1^ 2, while that in the side-stream anaerobic digestion liquor varies from 500 to 1500 mg N L^−1^ 3. The combination of partial nitritation and anaerobic ammonium oxidation (Anammox) has been proposed as an ideal treatment process and practiced to achieve high-level nitrogen removal from the anaerobic digestion liquor^4^,^5^,^6^,^7^,^8^,^9^,^10^. In this process, approximately half of ammonium is oxidized to nitrite by ammonium oxidizing bacteria (AOB) under aerobic conditions, while the formed nitrite and the remaining ammonium are autotrophically converted to nitrogen gas (with limited nitrate produced) by Anammox bacteria. Compared with the conventional nitrification-denitrification process, this autotrophic nitrogen removal reduces 60% of the aeration energy^11^,^12^.

In addition to ammonium, dissolved methane and sulfide are also commonly present in the anaerobic digestion liquor^13^. Their levels in the anaerobic digestion liquor depend on the wastewater source as well as the efficacy of the anaerobic treatment processes. During the partial nitritation process, dissolved methane in the anaerobic digestion liquor would be stripped to the atmosphere. Methane is a potent greenhouse gas with a warming potential about 34 times that of carbon dioxide (CO_2_) on a 100-year horizon^14^. Therefore, even low levels of methane emissions are unwanted. Sulfide is not only malodorous and corrosive^15^, but also toxic to human^16^ as well as a variety of microorganisms^17^. Therefore, the existence of dissolved methane and sulfide should be considered in assessing suitable downstream treatment technologies^1^. In other words, considerable efforts have to be dedicated to the management of dissolved methane and sulfide in the anaerobic digestion liquor.

The membrane biofilm reactor (MBfR) for simultaneous removal of ammonium, dissolved methane, and sulfide from the anaerobic digestion liquor is of significant interest because biofilms in the MBfR can retain microorganisms with distinct characteristics, and biomass can be naturally accumulated in the biofilm at different depths. By supplying oxygen through gas-permeable membranes while providing the anaerobic digestion liquor in the bulk liquid, the generated counter diffusion of gas and liquid substrates not only ensures a high gas transfer efficiency but also avoids methane stripping. The concurrent oxidation of ammonium, dissolved methane, and sulfide could be microbially catalyzed with oxygen as the electron acceptor. Moreover, a redox-stratified environment supporting both aerobic and anaerobic metabolisms across the biofilm layers would be created in such an MBfR. In addition to Anammox bacteria, the presence of nitrite and nitrate produced from ammonium oxidation together with the influent nutrients could stimulate the growth of denitrifying anaerobic methane oxidation (DAMO) microorganisms^18^ and sulfur oxidizing bacteria (SOB)^19^ within the biofilm.

Through using methane as the electron donor, DAMO archaea are capable of reducing nitrate to nitrite^20^ while DAMO bacteria are able to convert nitrite to nitrogen gas^21^. SOB can utilize reduced sulfur compounds (e.g., sulfide) as electron donor while using oxygen under aerobic conditions and/or nitrogen oxides under anaerobic conditions as electron acceptor for respiration^19^. Therefore, the coculture of AOB, Anammox bacteria, DAMO microorganisms, aerobic methane oxidizing bacteria (MOB), and SOB could be integrated in a single-stage MBfR to likely achieve the simultaneous removal of ammonium, dissolved methane, and sulfide from the anaerobic digestion liquor. A single-stage MBfR has been proposed by Chen *et al.*^22^ to achieve the simultaneous ammonium and dissolved methane removal from the side-stream anaerobic digestion liquor. However, to date no effort has yet been reported to investigate the feasibility of such an MBfR system for simultaneous removal of ammonium, dissolved methane, and sulfide, especially from the main-stream anaerobic digestion liquor.

Mathematical models can be used to study new biochemical processes and have been applied to assess emerging technologies as demonstrated previously^23^,^24^,^25^,^26^,^27^. In this work, a multi-species and multi-substrate biofilm model was developed to evaluate the conceptual feasibility of simultaneous ammonium, dissolved methane, and sulfide removal from both main-stream and side-stream anaerobic digestion liquors using single-stage MBfRs. A series of simulation scenarios concerning key operational parameters, i.e., influent surface loading (*L*_*IN*_ or hydraulic retention time (HRT)) and oxygen surface loading (*L*_*O*2_), was carried out through applying previously well-established species-specific biokinetics. The results of this work provide not only first insights into the selection pressures on microbial community development in the MBfR biofilm which directly determines the system performance, but also useful information for the process design and control of such a new technology which may facilitate the plant-wide sustainable operation of wastewater treatment systems.

## Results and Discussion

### Simultaneous ammonium, dissolved methane, and sulfide removal

By applying the developed model detailed in Tables S1–S4 in the Supporting Information (SI), the influent and effluent characteristics and system performance of the steady-state main-stream and side-stream MBfRs under the operational conditions of Scenario 0 (Table 1) are simulated and then shown in Table S5 in the SI. For the main-stream MBfR, the influent ammonium (50 g N m^−3^), dissolved methane (50 g COD m^−3^), and sulfide (30 g S m^−3^) are significantly removed, with concentrations of 3.6 g N m^−3^, 0.7 g COD m^−3^, and 0.3 g S m^−3^ in the effluent, respectively. Neither nitrite nor nitrate is produced, while the formed sulfate is dominant in the effluent. The resulting total nitrogen (TN), dissolved methane, and sulfide removal efficiencies are 92.8%, 98.6%, and 99.0%, respectively. Comparatively, the TN, dissolved methane, and sulfide removal efficiencies reach up to 96.8%, 99.8%, and 99.7%, respectively, for the side-stream MBfR with the influent ammonium, dissolved methane, and sulfide concentrations of 1000 g N m^−3^, 100 g COD m^−3^, and 30 g S m^−3^, respectively. The effluent contains mainly ammonium (7.2 g N m^−3^), nitrite (6.1 g N m^−3^), nitrate (19.1 g N m^−3^), and sulfate (29.9 g S m^−3^). The high-level removal demonstrates the feasibility of the proposed MBfR to achieve simultaneous ammonium, dissolved methane, and sulfide removal from both main-stream and side-stream anaerobic digestion liquors.

### Microbial community structure and substrate profiles in the biofilm

**Figure 1:**
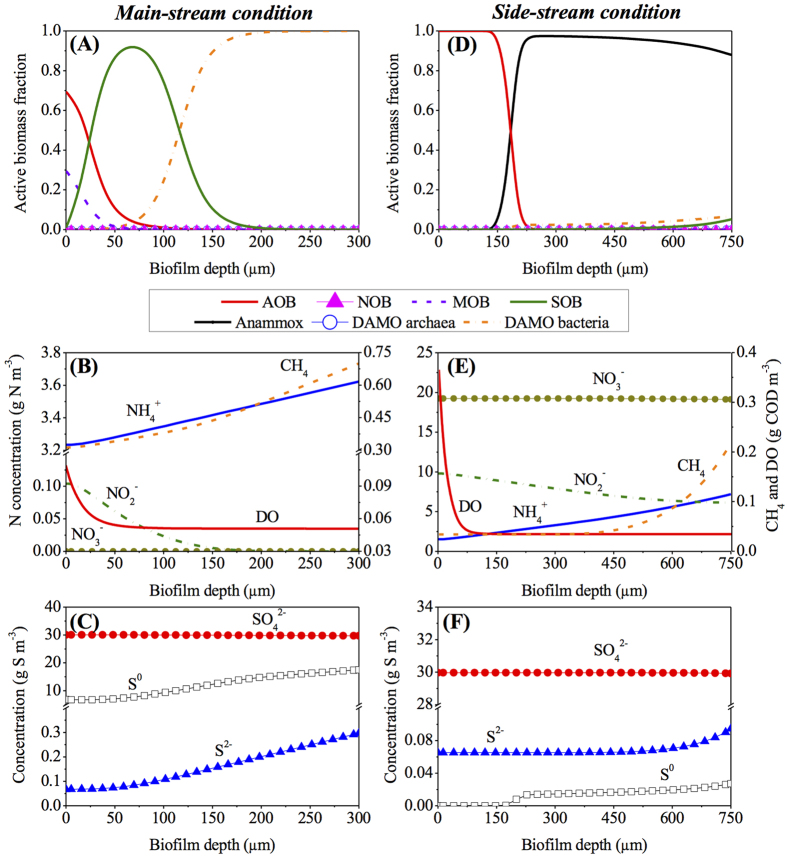
Modeling results of the MBfR for the simultaneous removal of ammonium, dissolved methane, and sulfide from main-stream (**A**–**C**) and side-stream (**D**–**F**) anaerobic digestion liquor based on Scenario 0 in Table 1 (depth zero represents the membrane surface at the base of the biofilm): (**A**,**D**) Microbial population distribution; (**B**,**E**) distribution profiles of nitrogen species, methane, and dissolved oxygen; and (**C**,**F**) distribution profiles of sulfur species.

The steady-state microbial population distribution and the concentration profiles of substrates and products within the biofilms of main-stream and side-stream MBfRs under the operational conditions of Scenario 0 (Table 1) are shown in Fig. 1. In the main-stream MBfR biofilm, AOB are dominant at the base of the biofilm from 0 to 50 μm in symbiosis with MOB, while DAMO bacteria dominate the outer layer of the biofilm from 100 μm to 300 μm. SOB are the dominant species in the middle layer but widely distributed from 0 to 200 μm (see Fig. 1A). The corresponding substrate and product profiles within the biofilm are shown in Fig. 1B,C. Dissolved oxygen (DO) decreases in the inner layer of the biofilm from 0 to 50 μm due to its consumption by AOB and MOB, which is consistent with the distribution of AOB and MOB in Fig. 1A. NH_4_^+^ concentration decreases from the surface to the base of the biofilm, while NO_2_^−^ drops below 0.01 g N m^−3^ at the biofilm thickness of over 150 μm. CH_4_ decreases continuously towards the base of the biofilm as the result of its consumption by DAMO bacteria in the outer layer and MOB in the inner layer of the biofilm. NO_3_^−^ is not present throughout the biofilm owing to the absence of nitrite oxidizing bacteria (NOB) and Anammox bacteria under the simulation conditions of Scenario 0, which thus constrains the growth of DAMO archaea (Fig. 1A). S^2−^ and hence S gradually decrease from the surface to the base of the biofilm due to the consumption by SOB, while the produced SO_4_^2−^ remains around 30.0 g S m^−3^ throughout the biofilm (Fig. 1C).

**Figure 2:**
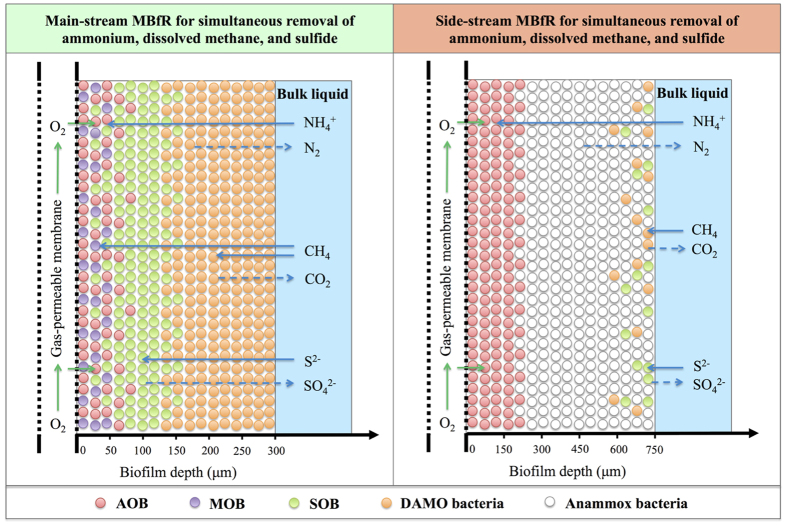
Conceptual substrate fluxes and microbial distribution of the main-stream and side-stream MBfRs for the simultaneous ammonium, dissolved methane, and sulfide removal from anaerobic digestion liquor.

A different microbial distribution is observed within the side-stream MBfR biofilm, as shown in Fig. 1D. AOB dominate the inner layer of the biofilm from 0 to 175 μm. In contrast, Anammox bacteria are dominant in the outer layer of the biofilm from 175 μm to 750 μm, with the coexistence of small fractions of SOB and DAMO bacteria (both <0.1) at the surface of the biofilm. Figure 1E,F illustrates the associated substrate profiles within the side-stream MBfR biofilm. DO quickly decreases in the inner layer of the biofilm as the result of its consumption by AOB. Mainly due to the contribution from Anammox bacteria, NO_2_^−^ decreases from the base towards the surface of the biofilm, while an opposite trend is observed for NH_4_^+^ (Fig. 1E). CH_4_ is consumed by DAMO bacteria and therefore quickly decreases in the outer layer of the biofilm. Because of the extremely low fraction of DAMO archaea in the biofilm under the simulation conditions of Scenario 0 (Fig. 1D), NO_3_^−^ produced by Anammox bacteria remains almost constant at 19.1 g N m^−3^ across the biofilm range (Fig. 1E). S^2−^ and hence S slightly decrease at the surface due to the consumption by a small number of SOB therein, while the produced SO_4_^2−^ remains constant throughout the biofilm (Fig. 1F). These observations reveal the specific microbial stratification in the biofilms of main-stream and side-stream MBfRs to achieve simultaneous ammonium, dissolved methane, and sulfide removal, which is due to the counter diffusion of gaseous oxygen from the membrane lumen and dissolved substrates from the bulk liquid, as shown in Fig. 2.

### Sensitivity analysis

The simulation results might be dependent on the proper selection of model parameter values. Therefore, considering the various sources of model parameters in this work, a sensitivity analysis (refer to the SI for detailed methodology) was conducted to evaluate the model structure and to investigate the most determinant biokinetic parameters on the system performance of the proposed main-stream and side-stream MBfRs in terms of TN, dissolved methane, and sulfide removal using the AQUASIM built-in algorithms, with results shown in Figures S1 and S2 in the SI, respectively. Specifically, as demonstrated in Figure S1, the TN removal efficiency of the main-stream MBfR is most sensitive to AOB-related biokinetic parameters, which represents the decisive role of AOB in the nitrogen removal of the main-stream MBfR. In contrast, the dissolved methane and sulfide removal efficiencies are most dependent on MOB-related parameters. These parameters affect the microbial competition between MOB and DAMO bacteria for the influent methane supply, which directly determines the dissolved methane removal. The sulfide removal is consequently affected due to the competition of SOB against DAMO bacteria for the availability of intermediate nitrite produced by AOB as electron acceptor.

For the side-stream MBfR, the TN and sulfide removal efficiencies are more sensitive to model parameters, compared to the dissolved methane removal efficiency (Figure S2). On the whole, the most sensitive parameters for the performance are the yield coefficients for Anammox bacteria (*Y*_*An*_) and AOB (*Y*_*AOB*_), and maximum growth rates of DAMO archaea (*μ*_*Da*_), DAMO bacteria (*μ*_*Db*_) and Anammox bacteria (*μ*_*An*_). These parameters directly regulate the microbial community structure in the side-stream MBfR biofilm, which therefore determines the system performance.

It should be noted that the system performance of both main-stream and side-stream MBfRs is relatively less sensitive to the parameters related to SOB under the studied operational conditions (Figures S1 and S2). This confirms the rationality and applicability of the obtained simulation trends despite the slight difference in the environments for SOB between the source studies^28^,^29^ and this work. Technically in the future application of the model, it is not practical to measure all of the numerous biokinetic parameters involved. In fact, accurate determination of those particularly sensitive to the performance of main-stream or side-stream MBfR (as discussed herein) in combination with reported values of other parameters could significantly reduce the workload while generating reliable results. Nevertheless, the model would be greatly improved while the simulation outcomes further validated upon the advent of the practical demonstration of the proposed MBfRs.

### Impact of influent surface loading on the MBfR

**Figure 3:**
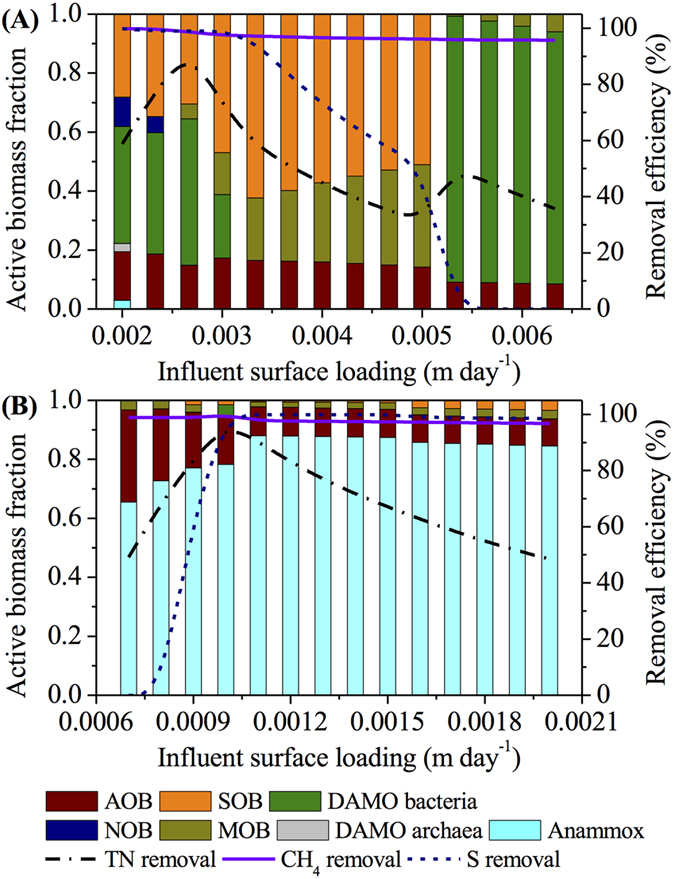
Modeling results of the effects of influent surface loading (*L*_*IN*_) on the main-stream (**A**) and side-stream (**B**) MBfRs based on Scenario 1 in Table 1.

The impact of *L*_*IN*_ on the TN, dissolved methane, and sulfide removal, and the microbial abundance in the biofilm of the main-stream MBfR (Scenario 1 in Table 1) is delineated in Fig. 3A. With the increase of *L*_*IN*_ to 0.0027 m d^−1^, the TN removal efficiency increases to the maximum of 92.8% while the sulfide removal efficiency stays above 99.0%. Further increase in *L*_*IN*_ results in the decrease of the TN and sulfide removal efficiencies, reaching 31.2% and 50.9% at *L*_*IN*_ of 0.005 m d^−1^, respectively. When *L*_*IN*_ exceeds 0.005 m d^−1^, the TN removal efficiency slightly recovers to 50.3% at *L*_*IN*_ of 0.0053 m d^−1^ but shows a downward trend thereafter. In contrast, the sulfide removal efficiency quickly drops to zero at *L*_*IN*_ of over 0.0053 m d^−1^. Across the whole range of *L*_*IN*_ studied, the dissolved methane removal efficiency only slightly drops from 99.9% at *L*_*IN*_ of 0.002 m d^−1^ to 95.7% at *L*_*IN*_ of 0.0063 m d^−1^. The varied system performance of the main-stream MBfR is caused by the different microbial structure in the biofilm shaped by different *L*_*IN*_. As shown in Fig. 3A, AOB, DAMO bacteria, and SOB dominate the biofilm of the main-stream MBfR with small fractions of Anammox bacteria, DAMO archaea, and NOB at the relatively low *L*_*IN*_. With the increase of *L*_*IN*_ to 0.0027 m d^−1^, Anammox bacteria, DAMO archaea, and NOB disappear from the biofilm while the abundance of DAMO bacteria gradually increases, thus resulting in nearly unchanged dissolved methane and sulfide removal efficiencies but growing TN removal efficiency. At *L*_*IN*_ of 0.0027 m d^−1^ with the maximum TN removal efficiency of 92.8%, AOB, DAMO bacteria, MOB, and SOB coexist in the biofilm, accounting for 15%, 50%, 5%, and 30% of the active biomass, respectively. Further increase in *L*_*IN*_ favors the competition of MOB against DAMO bacteria over the increasing influent methane supply. Therefore, the abundance of DAMO bacteria significantly drops to around zero at *L*_*IN*_ of more than 0.0033 m d^−1^, while that of MOB gradually increases with *L*_*IN*_. When *L*_*IN*_ surpasses 0.005 m d^−1^, DAMO bacteria outcompete MOB for methane as electron donor and SOB for intermediate nitrite as electron acceptor. As the result, AOB and DAMO bacteria dominate the biofilm, leading to the sharp gain in the TN removal efficiency but abrupt drop of the sulfide removal efficiency to zero at *L*_*IN*_ of 0.0053 m d^−1^. At higher *L*_*IN*_ with excessive methane supply in the influent which benefits the growth of MOB, MOB reappear in the biofilm at the expense of the decreasing abundance of DAMO bacteria, which causes the decline in the TN removal efficiency (Fig. 3A).

In contrast, a different relationship as shown in Fig. 3B is observed between *L*_*IN*_ and the performance as well as the microbial community structure of the side-stream MBfR (Scenario 1 in Table 1). Due to the significantly higher ammonium concentration (1000 g N m^−3^) in the influent, compared to dissolved methane (100 g COD m^−3^) and sulfide (30 g S m^−3^), AOB and Anammox bacteria are dominant in the biofilm of the side-stream MBfR under all *L*_*IN*_ conditions studied. When *L*_*IN*_ is lower than 0.001 m d^−1^, MOB outcompete DAMO bacteria for the influent methane supply and therefore coexist with AOB and Anammox bacteria in the biofilm. With the increase in *L*_*IN*_, the active biomass fraction of Anammox bacteria increases, while that of AOB decreases, leading to the increasing TN removal efficiency. DAMO bacteria replace MOB at *L*_*IN*_ of 0.001 m d^−1^ as the result of the increased methane supply, giving rise to the maximum TN removal efficiency of 96.8%. Further increasing *L*_*IN*_ provides excessive ammonium and favors the competition of Anammox bacteria against DAMO bacteria for the availability of intermediate nitrite. Consequently, DAMO bacteria suddenly disappear while MOB recolonize the biofilm with AOB and Anammox bacteria. The TN removal efficiency therefore decreases continuously with *L*_*IN*_, reaching 48.5% at *L*_*IN*_ of 0.002 m d^−1^. SOB only exist in the biofilm at *L*_*IN*_ of over 0.0008 m d^−1^ with the active biomass fraction fluctuating between 1% and 3%. Accordingly, the sulfide removal efficiency quickly increases from zero at *L*_*IN*_ of 0.0008 m d^−1^ and remains above 99.0% at *L*_*IN*_ of more than 0.001 m d^−1^. Owing to the succession of MOB and DAMO bacteria in the biofilm (Fig. 3B), the dissolved methane removal efficiency is not significantly affected and stays above 97.0% across the range of *L*_*IN*_ studied.

### Impact of oxygen surface loading on the MBfR

**Figure 4:**
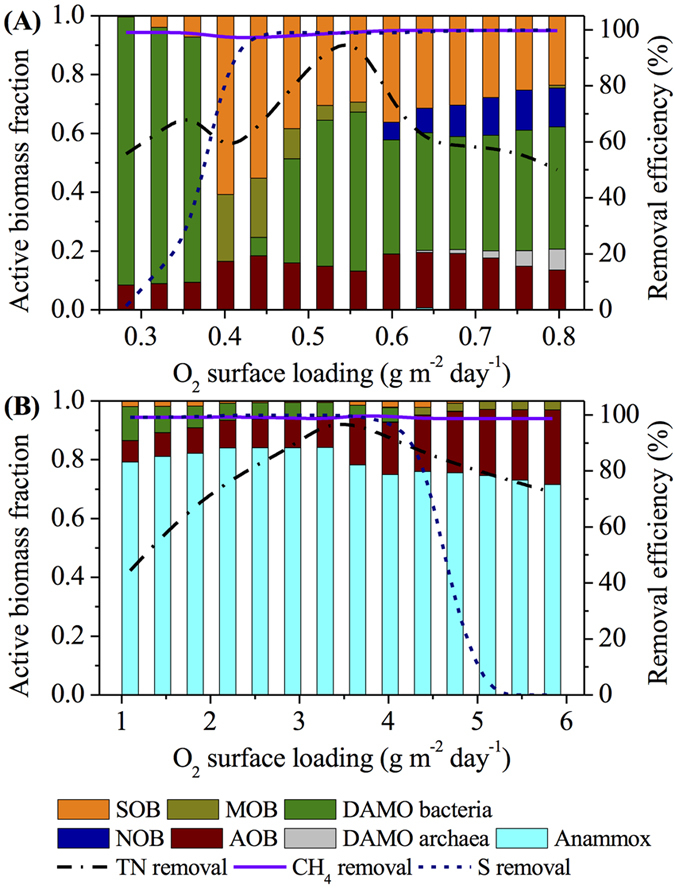
Modeling results of the effects of oxygen surface loading (*L*_*O*2_) on the main-stream (**A**) and side-stream (**B**) MBfRs based on Scsenario 2 in Table 1.

The dependence of the system performance and microbial community structure of the main-stream MBfR on *L*_*O*2_ (Scenario 2 in Table 1) is demonstrated in Fig. 4A. AOB and DAMO bacteria dominate the biofilm of the main-stream MBfR with a small fraction of SOB at *L*_*O*2_ of lower than 0.36 g m^−2^ d^−1^. With *L*_*O*2_ increasing from 0.28 to 0.36 g m^−2^ d^−1^, the abundance of SOB increases from less than 1% to 7% while that of DAMO bacteria decreases from 91% to 83%. The corresponding TN and sulfide removal efficiencies gradually increase from 55.7% and 1.4% to 71.4% and 27.3%, respectively. A higher *L*_*O*2_ of 0.40 g m^−2^ d^−1^ depresses the competition of DAMO bacteria against MOB over the influent methane supply and hence SOB over intermediate nitrite. As the result, DAMO bacteria are washed out from the biofilm while MOB and SOB act as the sole consumers of influent methane and intermediate nitrite, respectively. The corresponding TN removal efficiency drops to 55.3% while the sulfide removal efficiency quickly rises to 90.3% at *L*_*O*2_ of 0.40 g m^−2^ d^−1^. As *L*_*O*2_ further increases to 0.56 g m^−2^ d^−1^, the availability of intermediate nitrite favors the growth of DAMO bacteria again, resulting in the increasing abundance of DAMO bacteria but decreasing abundance of MOB and SOB in the biofilm. Thus, the TN removal efficiency increases to the maximum of 97.5% at *L*_*O*2_ of 0.56 g m^−2^ d^−1^. In contrast, the sulfide removal efficiency quickly jumps to 99.0% at *L*_*O*2_ of 0.44 g m^−2^ d^−1^ and remains almost unchanged thereafter. Further increasing *L*_*O*2_ stimulates the growth of NOB and hence DAMO archaea, leading to the consistent decline in the TN removal efficiency. Comparatively, no significant change is observed in the sulfide removal efficiency. Dissolved methane in the influent of the main-stream MBfR is alternately removed by DAMO bacteria and/or MOB under different *L*_*O*2_ conditions, resulting in the stable TN removal efficiency of above 99.0% across the range of *L*_*O*2_ studied (Fig. 4A).

Figure 4B shows the impact of *L*_*O*2_ on the system performance and microbial structure of the side-stream MBfR (Scenario 2 in Table 1). With the increasing *L*_*O*2_, the TN removal efficiency first increases from 44.4% to the maximum of 97.1% at *L*_*O*2_ of 3.29 g m^−2^ d^−1^, and gradually decreases thereafter, reaching 72.4% at *L*_*O*2_ of 5.84 g m^−2^ d^−1^. In contrast, the sulfide removal efficiency remains above 99.0% until *L*_*O*2_ reaches 3.65 g m^−2^ d^−1^ and then quickly drops to around zero at *L*_*O*2_ of over 5.11 g m^−2^ d^−1^. No significant change is observed in the dissolved methane removal efficiency, which stays around 99.0% across the whole range of *L*_*O*2_ studied. As demonstrated in Fig. 4B, the microbial community structure in the biofilm which is responsible for the shift in the performance of the side-stream MBfR changes with different *L*_*O*2_ conditions applied. AOB, Anammox bacteria, and DAMO bacteria jointly dominate the biofilm with a small fraction of SOB when *L*_*O*2_ is below 4.02 g m^−2^ d^−1^. Specially, the active biomass fractions of AOB and Anammox bacteria slightly increase with *L*_*O*2_, while that of DAMO bacteria decreases due to their intense competition against Anammox bacteria for intermediate nitrite. At *L*_*O*2_ of 3.29 g m^−2^ d^−1^ with the maximum TN removal efficiency of 97.1%, AOB, Anammox bacteria, DAMO bacteria, and SOB coexist in the biofilm of the side-stream MBfR, accounting for 12%, 83%, 4%, and 1% of the active biomass. Further increase in *L*_*O*2_ stimulates the growth of AOB, however, the growth of Anammox bacteria is limited due to finite NO_2_^−^/NH_4_^+^ mixture produced at the fixed influent surface loading of 0.001 m d^−1^. Therefore, the active biomass fraction of AOB increases while that of Anammox decreases. DAMO bacteria disappear from the biofilm at *L*_*O*2_ of more than 4.38 g m^−2^ d^−1^, due to their loss in the competition against MOB for the influent methane supply. Moreover, the growth of SOB is inhibited at *L*_*O*2_ of over 5.11 g m^−2^ d^−1^, as the result of high NO_2_^−^ accumulation under these *L*_*O*2_ conditions.

### Optimized operational conditions for the MBfR

Additional simulations (data not shown) based on the operational conditions of Scenario 0 demonstrate that a biofilm thickness of ≥150 μm and ≥450 μm is sufficient to achieve high-level simultaneous removal of ammonium, dissolved methane, and sulfide for the main-stream and side-stream MBfRs, respectively, while a thinner biofilm thickness would adversely affect the TN and sulfide removal. In contrast, the influent (containing ammonium, dissolved methane, and sulfide) and oxygen surface loadings jointly regulate the microbial community structure of the biofilm and thus significantly affect the overall performance of both main-stream and side-stream MBfRs as indicated in Figs 3 and 4. Therefore, Scenario 3 in Table 1 was designed to investigate the combined optimal operational conditions of main-stream and side-stream MBfRs in terms of *L*_*IN*_ and *L*_*O*2_ at a sufficient biofilm thickness with the former inversely proportional to and practically interpreted as HRT hereon.

**Figure 5:**
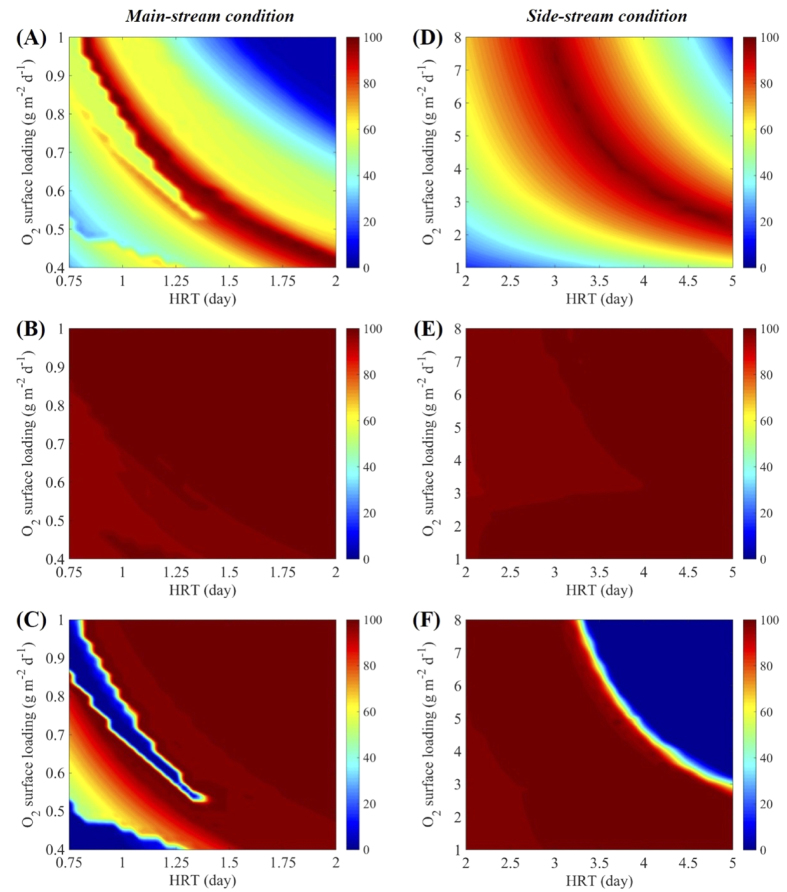
Modeling results of the performance of main-stream (**A–C**) and side-stream (**D–F**) MBfRs under different HRT and oxygen surface loading (*L*_*O*2_) conditions based on Scenario 3 in Table 1 in terms of TN removal (**A**,**D**), dissolved methane removal (**B**,**E**), and sulfide removal (**C**,**F**). The color scales represent removal efficiency in %.

**Figure 6:**
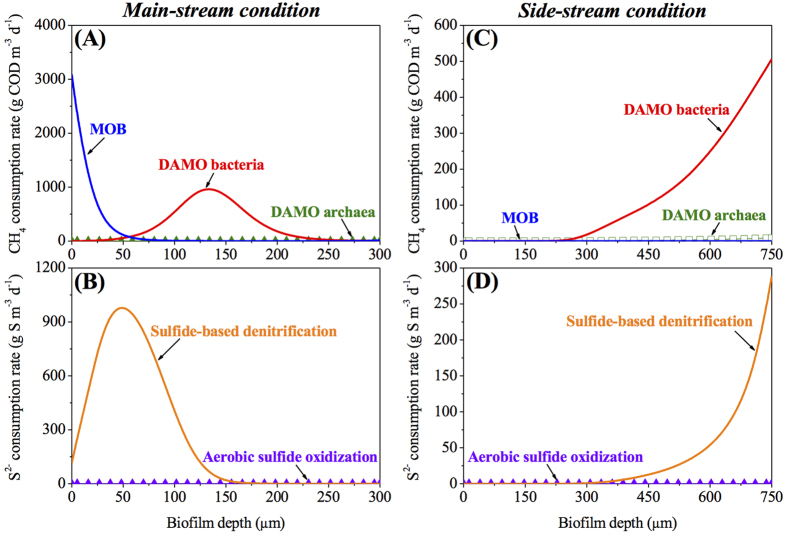
Species-specific dissolved methane and sulfide consumption rates within the biofilms of the main-stream MBfR (**A**,**B**) and the side-stream MBfR (**C**,**D**) under the optimal operational conditions. The applied influent surface loading (*L*_*IN*_), oxygen surface loading (*L*_*O*2_), and biofilm thickness (*L*_*f*_) for main-stream MBfR (**A**,**B**) are 0.0027 m d^−1^, 0.56 g m^−2^ d^−1^, and 300 μm, respectively. The applied influent surface loading (*L*_*IN*_), oxygen surface loading (*L*_*O*2_), and biofilm thickness (*L*_*f*_) for side-stream MBfR (**C**,**D**) are 0.001 m d^−1^, 3.65 g m^−2^ d^−1^, and 750 μm, respectively.

Figure 5A–C illustrates the TN, dissolved methane, and sulfide removal efficiencies, respectively, of the steady-state main-stream MBfR under the extensive simulation conditions (different combinations of HRT and *L*_*O*2_) of Scenario 3 in Table 1. For the main-stream MBfR, the simulated influent ammonium (50 g N m^−3^), dissolved methane (50 g COD m^−3^), and sulfide (30 g S m^−3^) are comparable quantitatively. Therefore, a short HRT intensifies the microbial interactions within the biofilm, e.g., the competition between AOB and Anammox bacteria for ammonium, the competition between MOB and DAMO microorganisms for methane, the competition between AOB, NOB, and MOB for oxygen, and the competition between DAMO bacteria, SOB, NOB, and Anammox bacteria for intermediate nitrite. Consequently, the TN and sulfide removal efficiencies are highly sensitive to *L*_*O*2_ when HRT is below 1.4 day, which corresponds to *L*_*IN*_ of over 0.0029 m d^−1^. Therein, a small change in *L*_*O*2_ would significantly alter the steady-state microbial community structure and hence affect the TN and sulfide removal efficiencies, as shown in Fig. 5A,C. In contrast, due to the alternating existence of DAMO microorganisms and MOB, no significant change is observed in the dissolved methane removal efficiency, which remains above 95.0% across the simulated ranges of HRT and *L*_*O*2_ (Fig. 5B). Despite the drastic variations in the TN and sulfide removal efficiencies at low HRTs, a distinct relationship is observed between the optimal HRT and *L*_*O*2_ for the high-level (>97.0%) simultaneous removal of ammonium, dissolved methane, and sulfide in the main-stream MBfR. As indicated in the dark red ridge-shape region in Fig. 5A, the optimal *L*_*O*2_ decreases with the increasing HRT. Under these optimal operating conditions, AOB, DAMO bacteria, MOB, and SOB coexist in the MBfR biofilm and cooperate to achieve the simultaneous removal of ammonium, dissolved methane, and sulfide from the main-stream anaerobic digestion liquor. As evidenced by the dissolved methane and sulfide consumption profiles within the main-stream MBfR biofilm under one optimal operational condition in Fig. 6A,B, approximately 50% of dissolved methane in the influent is consumed by DAMO bacteria to facilitate the nitrogen removal, while the remaining is oxidized by MOB (Fig. 6A). In contrast, nearly 100% of sulfide in the influent is utilized as electron donor by SOB for denitrification (Fig. 6B).

Different from the main-stream MBfR, clear trends are observed for the side-stream MBfR concerning the joint effect of HRT and *L*_*O*2_ on the steady-state overall system performance under the extensive simulation conditions of Scenario 3 in Table 1. The dark red ridge-shape region in Fig. 5D represents the ranges of optimal HRT and *L*_*O*2_ for high-level (>97.0%) simultaneous removal of ammonium, dissolved methane, and sulfide in the side-stream MBfR. Specifically, the optimal *L*_*O*2_ increases with the decreasing HRT. AOB and Anammox bacteria dominate the biofilm and are the key contributors to the ammonium removal under these optimal conditions. Comparatively, the coexisting small fractions of DAMO bacteria and SOB are solely responsible for the dissolved methane and sulfide removal (as evidenced by the example case in Fig. 6C,D), respectively, both of which enhance the nitrogen removal. An excessive oxygen supply at a certain HRT or a prolonged HRT at a certain *L*_*O*2_ will significantly depress the growth of SOB, resulting in the failure of the sulfide removal efficiency (as demonstrated in the dark blue region in Fig. 5F).

From the perspective of system operation, HRT and *L*_*O*2_ should be controlled wisely for both main-stream and side-stream MBfRs based on the dark red ridge-shape regions in Fig. 5A,D, respectively. Applicable to both main-stream and side-stream MBfRs, the optimal HRT is inversely proportional to the corresponding *L*_*O*2_. A long HRT will reduce the handling capacity of the MBfR, while a high *L*_*O*2_ will increase the treatment cost. The specific trade-off warrants further study.

In addition to dissolved methane, the utilization of sulfide originally present in the anaerobic digestion liquor possesses significant advantages over previous treatment options, which mainly focus on nitrogen removal. Sulfide, which is undesirable and harmful to downstream processing if left untreated, could act as additional electron donor to promote the nitrogen removal, alleviating its dependence on Anammox and DAMO processes. Moreover, compared with the work of Chen *et al.*^22^, the approved feasibility and the obtained optimized conditions of the main-stream MBfR represent the first step towards in-depth investigations of this technology, which would in turn facilitate further implementation of the main-stream anaerobic digestion in pursuit of sustainable operation at WWTPs.

It is worth noting that the back diffusion of methane provided into the membrane lumen is not considered in this work. This is acceptable considering the high-level (>95.0%) dissolved methane removal over the studied ranges of operational conditions (see Fig. 5B,E), corresponding to a minor potential loss of methane via the membrane lumen. The back diffusion of dinitrogen produced is also not included in view of its insignificant role in affecting the system performance of the MBfRs.

### Potential impact of organic carbon on the MBfR

A small amount of organic carbon remaining in the anaerobic digestion liquor might induce the heterotrophic growth, which might potentially affect the microbial community structure and hence the system removal performance of the MBfR^22^. Therefore, an additional simulation scenario was conducted to test the growth of heterotrophic bacteria (HB)^30^,^31^ in the main-stream and side-stream MBfRs at an influent organic carbon concentration of 30 g COD m^−3^ and 100 g COD m^−3^, respectively, on the premise of Scenario 0 in Table 1.

**Figure 7:**
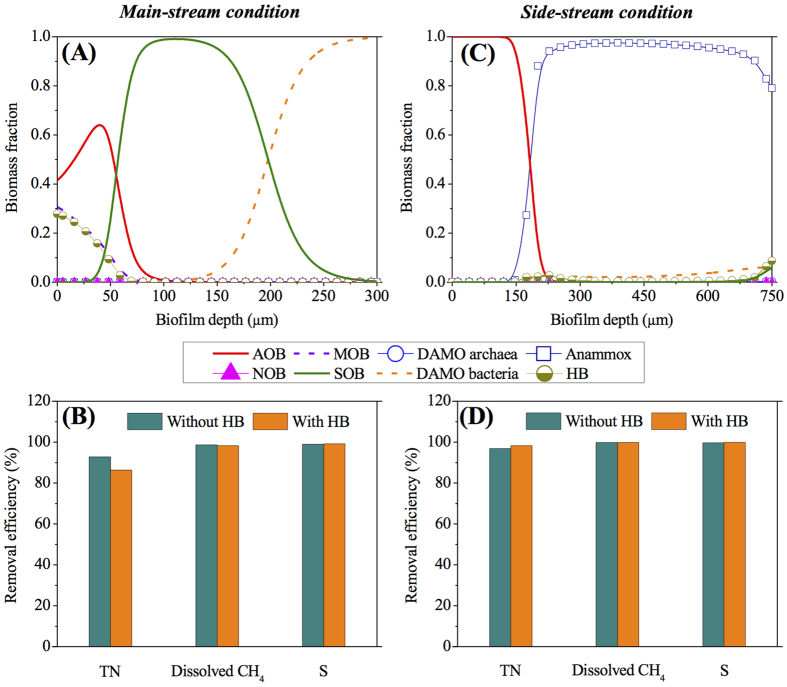
Modeling results of the MBfR for the simultaneous removal of ammonium, dissolved methane, and sulfide from main-stream (**A**,**B**) and side-stream (**C**,**D**) anaerobic digestion liquor in consideration of the potential existence of heterotrophic bacteria (HB) due to an influent organic carbon concentration of 30 g COD m^−3^ and 100 g COD m^−3^, respectively: (**A**,**C**) Microbial population distribution; and (**B**,**D**) TN, dissolved methane, and sulfide removal efficiencies with/without considering HB. The applied conditions are based on Scenario 0 in Table 1.

As indicated in Fig. 7A, HB grow in the inner biofilm layer of the main-stream MBfR, occupying around 8% of the total active biomass. Compared to Fig. 1A, the decreased abundance of AOB at the membrane surface results in 6% decrease in the TN removal efficiency, although no significant change has been observed in the dissolved methane and sulfide removal efficiencies (Fig. 7B). About 67% of the influent organic carbon is concurrently removed. In comparison, the influent organic carbon would only lead to a small fraction of heterotrophic growth (~1% of the total active biomass) in the biofilm of the side-stream MBfR (mainly at the biofilm surface) and a slight increase (~1%) in the TN removal efficiency without significant change in the dissolved methane and sulfide removal efficiencies (Fig. 7D), similar to Chen *et al.*^22^. Furthermore, around 97% of the influent organic carbon is consumed by heterotrophs. These findings prove the validity and effectiveness of the proposed MBfR to achieve simultaneous ammonium, dissolved methane, and sulfide removal with possible presence of organic carbon, due to its relatively minor impacts on the overall microbial community structure as well as the system removal performance under both main-stream and side-stream conditions (Fig. 7). However, efforts should be dedicated to minimizing the residual organic carbon in the main-stream anaerobic digestion liquor (i.e., maximizing the organic carbon conversion to methane in the main-stream anaerobic digester) in prior to its treatment via the proposed MBfR, which not only benefits the bioenergy recovery but also enhances the treatment efficiency of the proposed MBfR, especially the TN removal.

Apart from the organic carbon remaining in the anaerobic digestion liquor, the biomass decay products might also stimulate the heterotrophic growth to some extent. However, this type of heterotrophic growth is not included in the model, which is justified by the negligible impacts of the heterotrophic growth on the biomass decay products on the steady-state behaviour of a similar single-stage reactor coupling partial nitritation and Anammox^31^. In addition to organic carbon, other factors (e.g., pH and potential inhibition of sulfide) might also affect the microorganisms involved and hence the performance of the MBfR for simultaneous ammonium, dissolved methane, and sulfide removal from the anaerobic digestion liquors. Therefore, to render a more comprehensive understanding of the proposed technology, further investigations could probe into the roles of these factors as well as the experimental demonstration of the MBfR based on the information derived from this work.

## Materials and Methods

### MBfR

The simulated MBfR in this work has a working volume of 1 m^3^, including 0.96 m^3^ bulk volume outside the gas-permeable membranes and 0.04 m^3^ gas volume inside the membrane lumen. The total membrane surface area for oxygen supply and biofilm attachment is 240 m^2^, resulting in a surface to volume ratio of 250 m^2^ m^−3^. This reactor setup is similar to the ones used by Pellicer-Nacher *et al.*^32^ and Ni and Yuan^26^. Compressed air is supplied in flow-through mode to the membrane module, with the oxygen flux to the biofilm controlled through changing either the applied gas pressure or the gas flow rate into the membrane lumen. Based on the values reported in literature, the simulated ammonium, dissolved methane, and sulfide concentrations are typically set at 50 g N m^−3^ 33,34,35, 50 g COD m^−3^ 33,36, and 30 g S m^−3^ 13,33, respectively, for the main-stream anaerobic digestion liquor, while those are 1000 g N m^−3^ 25, 100 g COD m^−3^ 25, and 30 g S m^−3^ 13, respectively, for the side-stream anaerobic digestion liquor. The influent flow rate is varied to regulate the influent surface loading which also corresponds to HRT.

### Biofilm model

To simulate the bioconversion processes and microbial community structure in the MBfR, a multi-species and multi-substrate one-dimensional biofilm model is constructed and employed on the software AQUASIM 2.1d^37^. The MBfR is modeled as composed of two different compartments: a completely mixed gas compartment (representing the membrane lumen operated as flowthrough) and a biofilm compartment, containing the biofilm and bulk liquid. The gas compartment is connected to the base of the biofilm via a diffusive link. The gaseous concentration of oxygen in the gas compartment is determined by the applied gas pressure along with the gas flow rate. The oxygen flux from the gas to the biofilm matrix compartment through the membrane is modeled based on Henry’s law in consideration of an overall mass transfer coefficient of oxygen^24^.

Biofilm structures are represented as a continuum without considering diffusive mass transport of biomass in the biofilm matrix. The steady-state biofilm thickness is established by controlling the detachment using a global detachment velocity, i.e., *u*_*de*_ (μm d^−1^), in model simulations. The surface detachment velocity is modeled based on the biofilm growth velocity, *u*_*F*_ (μm d^−1^), the biofilm thickness, *L*_*f*_(μm), and the desired mean biofilm thickness, *L*_*f*,*mean*_ (μm), as follows: *u*_*de*_ = *u*_*F*_ × (*L*_*f*_/*L*_*f*,*mean*_)^10^. The composition of solids detached from the biofilm conforms to their composition at the biofilm surface. The detached particulates are assumed to be washed out of the system with the effluent, and no re-attachment of detached particulates is considered in the model. The water fraction of the biofilm matrix is kept constant at 0.75, while the biomass density is 50000 g COD m^−3^ 38. Similar to Terada *et al.*^24^, mass transfer coefficients for soluble components in the liquid phase of the biofilm are set at 0.8-fold of the values in water. Parameters regarding the mass transfer coefficients for nitrogen species and oxygen are selected according to Hao *et al.*^39^. The mass transfer coefficients for sulfate and sulfide are taken from Stewart^40^, while that for methane is adopted from Cussler^41^.

### Biological model

The components, kinetics, and stoichiometry of the developed biochemical reaction model at a temperature of about 25 °C and a neutral pH are summarized in Tables S1–S3 in the SI. The biochemical reaction model describes the metabolic relationships among nine particulate species, namely AOB (with concentration denoted as *X*_*AOB*_), NOB (*X*_*NOB*_), DAMO archaea (*X*_*Da*_), DAMO bacteria (*X*_*Db*_), Anammox bacteria (*X*_*An*_), SOB (*X*_*SOB*_), MOB (*X*_*MOB*_), inert biomass (*X*_*I*_), and elemental sulfur (*X*_*S*_), and stoichiometric relationships among eight soluble species, namely ammonium (*S*_*NH4*_), nitrite (*S*_*NO2*_), nitrate (*S*_*NO3*_), dinitrogen (*S*_*N*2_), methane (*S*_*CH4*_), oxygen (*S*_*O2*_), sulfide (*S*_*S*_), and sulfate (*S*_*SO4*_). For simplification, one population group is applied to represent SOB, which are capable of using oxygen, nitrite, or nitrate as electron acceptor, with electrons derived from oxidation of either sulfide or elemental sulfur. The possible pathway of sulfate reduction by Anammox bacteria is not included, in view of its relatively slow rate compared to the dominant conventional metabolisms of Anammox bacteria^42^.

Both growth and decay processes are considered for each species. Kinetic control of all the microbial reaction rates is described by the Monod equation, with each reaction rate modeled by an explicit function considering all substrates involved (Table S2 in the SI). The reported well-established parameter values that have been verified experimentally are used in this simulation study to characterize the metabolisms of nitrifying bacteria^24^,^43^, Anammox bacteria^38^,^39^,^44^,^45^, DAMO microorganisms^18^,^23^,^46^,^47^, SOB^28^,^29^,^48^, and MOB^49^. In particular, considering the reported multiple metabolic pathways of SOB^19^, biological conversions of sulfur species involving different electron donors (sulfide and elemental sulfur) and electron acceptors (oxygen, nitrate, and nitrite) are modeled comprehensively, as shown in processes 13–17 in Tables S2 and S3 (SI). The potential inhibition effect of sulfide on Anammox bacteria is not incorporated into this model, considering the contradictory conclusions reported^50^. In addition, the applied sulfide concentration of 30 mg L^−1^ in this work is much lower than the possible sulfide inhibition levels^50^,^51^ and thus might not exert a significant effect on Anammox. However, the model could be readily expanded or modified to include this impact if sulfide inhibition becomes important in the system. Table S4 in the SI shows the definitions, values, units, and sources of all parameters used in the biochemical reaction model.

### Simulation scenarios

Four different scenarios are considered for both main-stream and side-stream MBfRs in this work, as detailed in Table 1. The conditions for the first simulation scenario (Scenario 0 of Table 1) are selected in order to clearly and representatively demonstrate the mechanisms behind the system performance, which is realized through generating depth profiles of substrates (including nitrogen species, DO, dissolved methane, and sulfur species) and microbial community distribution in the MBfR biofilm. *L*_*IN*_, *L*_*O*2_, and *L*_*f*_ are 0.0027 m d^−1^ (i.e., an HRT of 1.5 days), 0.52 g m^−2^ d^−1^, and 300 μm for the main-stream MBfR, respectively, while 0.001 m d^−1^ (i.e., an HRT of 4 days), 3.65 g m^−2^ d^−1^, and 750 μm for the side-stream MBfR, respectively. Scenarios 1 and 2 in Table 1 investigate the effects of *L*_*IN*_ and *L*_*O*2_, respectively, on the removal efficiencies of ammonium, dissolved methane, and sulfide and the microbial community structure of main-stream and side-stream MBfR biofilms at steady state. The operational parameters for simulation are chosen systematically over wide ranges of *L*_*IN*_ (main-stream condition: 0.002–0.0063 m d^−1^, side-stream condition: 0.0007–0.002 m d^−1^) and *L*_*O*2_ (main-steam condition: 0.28–2 g m^−2^ d^−1^, side-steam condition: 1–5.84 g m^−2^ d^−1^). Scenario 3 in Table 1 examines the combined impact of *L*_*IN*_ and *L*_*O*2_ on the process performance and optimizes the operation of main-stream and side-stream MBfRs to achieve the simultaneous removal of ammonium, dissolved methane, and sulfide.

For each simulation scenario, the initial concentrations of all soluble components are assumed to be zero in the biofilm and in the bulk liquid. An average biofilm thickness is applied in the model without consideration of its variation with locations. All simulations assume an initial biofilm thickness of 20 μm. Compared to the side-stream MBfR, the main-stream MBfR usually undergoes a shorter HRT, giving rise to a greater shear force. Therefore, a lower steady-state biofilm thickness is used to simulate the main-stream MBfR (i.e., 300 μm) while a higher steady-state biofilm thickness is considered for the side-stream MBfR (i.e., 750 μm, same as Chen *et al.*^22^). Simulations are typically run for up to 1000 days to reach steady-state conditions in terms of effluent quality, biofilm thickness, and biomass compositions in biofilm. The steady-state TN, dissolved methane, and sulfide removal efficiencies are used to evaluate the performance of the MBfR.

## Additional Information

**How to cite this article**: Chen, X. *et al.* Model-Based Feasibility Assessment of Membrane Biofilm Reactor to Achieve Simultaneous Ammonium, Dissolved Methane, and Sulfide Removal from Anaerobic Digestion Liquor. *Sci. Rep.* **6**, 25114; doi: 10.1038/srep25114 (2016).

## Supplementary Material

Supporting Information

## Figures and Tables

**Table 1:** An overview of the simulation scenarios for the reported results.

Scenarios		Simulation conditions	Variable conditions
**Scenario 0** Standard simulation	Main-Stream	*S*_NH4_ = 50 g N m^−3^	
		*S*_CH4_ = 50 g COD m^−3^	
		*S*_S_ = 30 g S m^−3^	
		*L*_IN_ = 0.0027 m d^−1^	
		*L*_O2_ = 0.52 g m^−2^ d^−1^	
		*L*_f_ = 300 μm	
	Side-Stream	*S*_NH4_ = 1000 g N m^−3^	
		*S*_CH4_ = 100 g COD m^−3^	
		*S*_S_ = 30 g S m^−3^	
		*L*_IN_ = 0.001 m d^−1^	
		*L*_O2_ = 3.65 g m^−2^ d^−1^	
		*L*_f_ = 750 μm	
**Scenario 1** Effect of *L*_IN_	Main-Stream	*S*_NH4_ = 50 g N m^−3^	*L*_IN_ = 0.002–0.0063 m d^−1^
		*S*_CH4_ = 50 g COD m^−3^	
		*S*_S_ = 30 g S m^−3^	
		*L*_O2_ = 0.52 g m^−2^ d^−1^	
		*L*_f_ = 300 μm	
	Side-Stream	*S*_NH4_ = 1000 g N m^−3^	*L*_IN_ = 0.0007–0.002 m d^−1^
		*S*_CH4_ = 100 g COD m^−3^	
		*S*_S_ = 30 g S m^−3^	
		*L*_O2_ = 3.65 g m^−2^ d^−1^	
		*L*_f_ = 750 μm	
**Scenario 2** Effect of *L*_O2_	Main-Stream	*S*_NH4_ = 50 g N m^−3^	*L*_O2_ = 0.28–0.8 g m^−2^ d^−1^
		*S*_CH4_ = 50 g COD m^−3^	
		*S*_S_ = 30 g S m^−3^	
		*L*_IN_ = 0.0027 m d^−1^	
		*L*_f_ = 300 μm	
	Side-Stream	*S*_NH4_ = 1000 g N m^−3^	*L*_O2_ = 1–5.84 g m^−2^ d^−1^
		*S*_CH4_ = 100 g COD m^−3^	
		*S*_S_ = 30 g S m^−3^	
		*L*_IN_ = 0.001 m d^−1^	
		*L*_f_ = 750 μm	
**Scenario 3** Combined effect of *L*_IN_ and *L*_O2_	Main-Stream	*S*_NH4_ = 50 g N m^−3^	*L*_IN_ = 0.002–0.0053 m d^−1^
		*S*_CH4_ = 50 g COD m^−3^	HRT = 0.75–2 day
		*S*_S_ = 30 g S m^−3^	*L*_O2_ = 0.4–1 g m^−2^ d^−1^
		*L*_f_ = 300 μm	
	Side-Stream	*S*_NH4_ = 1000 g N m^−3^	*L*_IN_ = 0.0008–0.002 m d^−1^
		*S*_CH4_ = 100 g COD m^−3^	HRT*=* 2–5 day
		*S*_S_ = 30 g S m^−3^	*L*_O2_ = 1–8 g m^−2^ d^−1^
		*L*_f_ = 750 μm	
