# Supplementary material for: Model-Based Feasibility Assessment of Membrane Biofilm Reactor to Achieve Simultaneous Ammonium, Dissolved Methane, and Sulfide Removal from Anaerobic Digestion Liquor
Source: Sci Rep. 2016 Apr 26;6:25114. doi: 10.1038/srep25114 (PMC4844991; doi:10.1038/srep25114)
Supplement: Supporting Information [file srep25114-s1.pdf]

Supporting Information

Model-Based Feasibility Assessment of Membrane Biofilm Reactor to Achieve Simultaneous Ammonium, Dissolved Methane, and Sulfide Removal from Anaerobic Digestion Liquor

Xueming Chen, Yiwen Liu, Lai Peng, Zhiguo Yuan, Bing-Jie Ni*

Advanced Water Management Centre, The University of Queensland, St. Lucia, Brisbane, QLD 4072, Australia

*Corresponding author:

Dr. Bing-Jie Ni, P +61 7 3346 3230; F +61 7 3365 4726; E-mail b.ni@uq.edu.au

The following are included as supporting information for this paper:

Methodology for sensitivity analysis

A sensitivity analysis was performed using AQUASIM to test the sensitivities of the parameters concerned to the model output. The following "absolute-relative" sensitivity function is used:

$$\delta_{yp}^{\alpha r} = p\frac{\partial y}{\partial p}$$

where $\delta_{yp}^{\alpha r}$ is the sensitivity analytical output to a model parameter, y is an arbitrary value related to a model variable calculated with AQUASIM, and p is a model parameter. The function measures the absolute change of y for a 100% change in p. In this work, the system performance of both main-stream and side-stream MBfRs, including TN, dissolved CH_4, and S^{2-} removal efficiencies, is applied as the model output. All the kinetic and stoichiometric parameters in the model are involved in the sensitivity analysis, with their base values to initiate the procedure shown in Table S4. Only the positive values of $\delta_{yp}^{\alpha r}$ are loaded for the sensitivity analysis, with the detailed calculated results shown in Figures S1 and S2.

Table S1. Definition of Components in the Model

Number	Component	Definition	Unit
**Model dissolved components**			
1	S_{NH4}	Ammonium nitrogen	g N m^{-3}
2	S_{NO2}	Nitrite nitrogen	g N m^{-3}
3	S_{NO3}	Nitrate nitrogen	g N m^{-3}
4	S_{N2}	Dinitrogen	g N m^{-3}
5	S_{CH4}	Dissolved methane	g COD m^{-3}
6	S_{S}	Sulfide	g S m^{-3}
7	S_{SO4}	Sulfate	g S m^{-3}
8	S_{O2}	Dissolved oxygen	g O_2 m^{-3}
**Model particulate components**			
9	X_{AOB}	Aerobic ammonium oxidizing bacteria	g COD m^{-3}
10	X_{NOB}	Nitrite oxidizing bacteria	g COD m^{-3}
11	X_{Da}	Denitrifying anaerobic methane oxidizing archaea	g COD m^{-3}
12	X_{Db}	Denitrifying anaerobic methane oxidizing bacteria	g COD m^{-3}
13	X_{An}	Anaerobic ammonium oxidizing bacteria	g COD m^{-3}
14	X_{SOB}	Sulfur oxidizing bacteria	g COD m^{-3}
15	X_{MOB}	Aerobic methane oxidizing bacteria	g COD m^{-3}
16	X_{I}	Inert, non-biodegradable organics	g COD m^{-3}
17	X_{S}	Elemental sulfur	g S m^{-3}

Table S2. Process Kinetic Rate Equations for the Biological Reaction Model

Process	Kinetics rates expressions
Ammonium oxidizing bacteria (AOB)	
1. Growth of AOB	$\mu_{AOB}\frac{S_{NH4}}{S_{NH4}+K_{NH4}^{AOB}}\frac{S_{O2}}{S_{O2}+K_{O2}^{AOB}}X_{AOB}$
2. Decay of AOB	$b_{AOB}X_{AOB}$
Nitrite oxidizing bacteria (NOB)	
3. Growth of NOB	$\mu_{NOB}\frac{S_{NO2}}{S_{NO2}+K_{NO2}^{NOB}}\frac{S_{O2}}{S_{O2}+K_{O2}^{NOB}}X_{NOB}$
4. Decay of NOB	$b_{NOB}X_{NOB}$
DAMO archaea	
5. Growth of DAMO archaea	$\mu_{Da}\frac{S_{NO3}}{S_{NO3}+K_{NO3}^{Da}}\frac{S_{CH4}}{S_{CH4}+K_{CH4}^{Da}}\frac{K_{O2}^{Da}}{S_{O2}+K_{O2}^{Da}}\frac{K_{I,NO2}^{Da}}{S_{NO2}+K_{I,NO2}^{Da}}X_{Da}$
6. Decay of DAMO archaea	$b_{Da}X_{Da}$
DAMO bacteria	
7. Growth of DAMO bacteria	$\mu_{Db}\frac{S_{NO2}}{S_{NO2}+K_{NO2}^{Db}}\frac{S_{CH4}}{S_{CH4}+K_{CH4}^{Db}}\frac{K_{O2}^{Db}}{S_{O2}+K_{O2}^{Db}}\frac{K_{I,NO2}^{Db}}{S_{NO2}+K_{I,NO2}^{Db}}X_{Db}$
8. Decay of DAMO bacteria	$b_{Db}X_{Db}$
Anaerobic ammonium oxidizing bacteria (Anammox)	
9. Growth of Anammox	$\mu_{An}\frac{S_{NO2}}{S_{NO2}+K_{NO2}^{An}}\frac{S_{NH4}}{S_{NH4}+K_{NH4}^{An}}\frac{K_{O2}^{An}}{S_{O2}+K_{O2}^{An}}\frac{K_{I,NO2}^{An}}{S_{NO2}+K_{I,NO2}^{An}}X_{An}$
10. Decay of Anammox	$b_{An}X_{An}$
Methane oxidizing bacteria (MOB)	
11. Growth of MOB	$\mu_{MOB}\frac{S_{CH4}}{S_{CH4}+K_{CH4}^{MOB}}\frac{S_{O2}}{S_{O2}+K_{O2}^{MOB}}X_{MOB}$
12. Decay of MOB	$b_{MOB}X_{MOB}$
Sulfur oxidizing bacteria (SOB)	
13. Aerobic sulfide oxidization (R1)	$\mu_{SOB}^{R1}\frac{S_S}{S_S+K_S^{R1}}\frac{S_{O2}}{S_{O2}+K_{O2}^{R1}}X_{SOB}$
14. Sulfide-based nitrate-dependent denitrification (R2)	$\mu_{SOB}^{R2}\frac{S_S}{S_S+K_S^{R2}+(S_S)^2/K_{I,S}^{R2}}\frac{S_{NO3}}{S_{NO3}+K_{NO3}^{R2}}\frac{K_{I,NO2}^{R2}}{S_{NO2}+K_{I,NO2}^{R2}}X_{SOB}$
15. Sulfide-based nitrite-dependent denitrification (R3)	$\mu_{SOB}^{R3}\frac{S_S}{S_S+K_S^{R3}+(S_S)^2/K_{I,S}^{R3}}\frac{S_{NO2}}{S_{NO2}+K_{NO2}^{R3}+(S_{NO2})^2/K_{I,NO2}^{R3}}X_{SOB}$
16. Sulfur-based nitrate-dependent denitrification (R4)	$\mu_{SOB}^{R4}\frac{X_S}{X_S+K_S^{R4}}\frac{S_{NO3}}{S_{NO3}+K_{NO3}^{R4}}X_{SOB}$
17. Sulfur-based nitrite-dependent denitrification (R5)	$\mu_{SOB}^{R5}\frac{X_S}{X_S+K_S^{R5}}\frac{S_{NO2}}{S_{NO2}+K_{NO2}^{R5}}X_{SOB}$
18. Decay of SOB	$b_{SOB}X_{SOB}$

Table S3. Stoichiometric Matrix for the Biological Reaction Model

Variable<br>Process	S_{NH4}<br>N	S_{NO2}<br>N	S_{NO3}<br>N	S_{N2}<br>N	S_{CH4}<br>COD	S_S<br>S	X_S<br>S	S_{SO4}<br>S	S_{O2}<br>O2	X_{AOB}<br>COD	X_{NOB}<br>COD	X_{Da}<br>COD	X_{Db}<br>COD	X_{An}<br>COD	X_{MOB}<br>COD	X_{SOB}<br>COD	X_I<br>COD
1	$-i_{NBM}-\frac{1}{Y_{AOB}}$	$\frac{1}{Y_{AOB}}$							$-\frac{3.43-Y_{AOB}}{Y_{AOB}}$	1							
2	$i_{NBM}-i_{NXI}*f_I$									-1							f_I
3	$-i_{NBM}$	$-\frac{1}{Y_{NOB}}$	$\frac{1}{Y_{NOB}}$						$-\frac{1.14-Y_{NOB}}{Y_{NOB}}$		1						
4	$i_{NBM}-i_{NXI}*f_I$										-1						f_I
5	$-i_{NBM}$	$\frac{1-Y_{Da}}{1.14Y_{Da}}$	$-\frac{1-Y_{Da}}{1.14Y_{Da}}$		$-\frac{1}{Y_{Da}}$							1					
6	$i_{NBM}-i_{NXI}*f_I$											-1					f_I
7	$-i_{NBM}$	$-\frac{1-Y_{Db}}{1.71Y_{Db}}$		$\frac{1-Y_{Db}}{1.71Y_{Db}}$	$-\frac{1}{Y_{Db}}$								1				
8	$i_{NBM}-i_{NXI}*f_I$												-1				f_I
9	$-i_{NBM}-\frac{1}{Y_{An}}$	$-\frac{1}{Y_{An}}-\frac{1}{1.14}$	$\frac{1}{1.14}$	$\frac{2}{Y_{An}}$										1			
10	$i_{NBM}-i_{NXI}*f_I$													-1			f_I
11	$-i_{NBM}$				$-\frac{1}{Y_{MOB}}$				$-\frac{1-Y_{MOB}}{Y_{MOB}}$						1		
12	$i_{NBM}-i_{NXI}*f_I$														-1		f_I
13	$-i_{NBM}$					$-\frac{1}{Y_{SOB}}$	$\frac{1}{Y_{SOB}}$		$-\frac{1-2Y_{SOB}}{2Y_{SOB}}$							1	
14	$-i_{NBM}$	$\frac{1-2Y_{SOB}}{2.29Y_{SOB}}$	$-\frac{1-2Y_{SOB}}{2.29Y_{SOB}}$			$-\frac{1}{Y_{SOB}}$	$\frac{1}{Y_{SOB}}$									1	
15	$-i_{NBM}$	$-\frac{1-2Y_{SOB}}{3.43Y_{SOB}}$		$\frac{1-2Y_{SOB}}{3.43Y_{SOB}}$		$-\frac{1}{Y_{SOB}}$	$\frac{1}{Y_{SOB}}$									1	
16	$-i_{NBM}$	$\frac{1.5-Y_{SOB}}{1.14Y_{SOB}}$	$-\frac{1.5-Y_{SOB}}{1.14Y_{SOB}}$				$-\frac{1}{Y_{SOB}}$	$\frac{1}{Y_{SOB}}$								1	
17	$-i_{NBM}$	$-\frac{1.5-Y_{SOB}}{1.71Y_{SOB}}$		$\frac{1.5-Y_{SOB}}{1.71Y_{SOB}}$			$-\frac{1}{Y_{SOB}}$	$\frac{1}{Y_{SOB}}$								1	
18	$i_{NBM}-i_{NXI}*f_I$															-1	f_I

Table S4. Stoichiometric and Kinetic Parameters of the Developed Model

Parameter	Definition	Value	Unit	Source
Stoichiometric parameters				
Y_{AOB}	Yield coefficient for AOB	0.150	g COD g^{-1} N	Wiesmann 1994
Y_{NOB}	Yield coefficient for NOB	0.041	g COD g^{-1} N	Wiesmann 1994
Y_{Da}	Yield coefficient for DAMO archaea	0.071	g COD g^{-1} COD	Chen et al. 2014
Y_{Db}	Yield coefficient for DAMO bacteria	0.055	g COD g^{-1} COD	Chen et al. 2014
Y_{An}	Yield coefficient for Anammox	0.159	g COD g^{-1} N	Strous et al. 1998
Y_{MOB}	Yield coefficient for MOB	0.19	g COD g^{-1} COD	Daelman et al. 2014
Y_{SOB}	Yield coefficient for SOB	0.128	g COD g^{-1} S	Xu et al. 2013
i_{NBM}	Nitrogen content of biomass	0.07	g N g^{-1} COD	Henze et al. 2000
i_{NXI}	Nitrogen content of X_I	0.02	g N g^{-1} COD	Henze et al. 2000
f_I	Fraction of X_I in biomass decay	0.10	g COD g^{-1} COD	Henze et al. 2000
Ammonium oxidizing bacteria (AOB)				
μ_{AOB}	Maximum growth rate of AOB	0.0854	h^{-1}	Wiesmann 1994
b_{AOB}	Decay rate coefficient of AOB	0.0054	h^{-1}	Wiesmann 1994
K_{NH4}^{AOB}	S_{NH4} affinity constant for AOB	2.4	g N m^{-3}	Wiesmann 1994
K_{O2}^{AOB}	S_{O2} affinity constant for AOB	0.6	g COD m^{-3}	Wiesmann 1994
Nitrite oxidizing bacteria (NOB)				
μ_{NOB}	Maximum growth rate of NOB	0.0604	h^{-1}	Wiesmann 1994
b_{NOB}	Decay rate coefficient of NOB	0.0025	h^{-1}	Wiesmann 1994
K_{NO2}^{NOB}	S_{NO2} affinity constant for NOB	5.5	g N m^{-3}	Wiesmann 1994
K_{O2}^{NOB}	S_{O2} affinity constant for NOB	2.2	g COD m^{-3}	Wiesmann 1994
DAMO archaea				
μ_{Da}	Maximum growth rate of DAMO archaea	0.00151	h^{-1}	Chen et al. 2014
b_{Da}	Decay rate coefficient of DAMO archaea	0.00018	h^{-1}	Chen et al. 2014
K_{NO3}^{Da}	S_{NO3} affinity constant for DAMO archaea	0.11	g N m^{-3}	Chen et al. 2014
K_{CH4}^{Da}	S_{CH4} affinity constant for DAMO archaea	0.0384	g COD m^{-3}	Raghoebarsing et al. 2006
K_{O2}^{Da}	S_{O2} inhibition constant for DAMO archaea	0.64	g COD m^{-3}	Lopes et al. 2011
$K_{I,NO2}^{Da}$	S_{NO2} inhibition constant for DAMO archaea	57.4	g N m^{-3}	He et al. 2013
DAMO bacteria				
μ_{Db}	Maximum growth rate of DAMO bacteria	0.0018	h^{-1}	Chen et al. 2014
b_{Db}	Decay rate coefficient of DAMO bacteria	0.00018	h^{-1}	Chen et al. 2014
K_{NO2}^{Db}	S_{NO2} affinity constant for DAMO bacteria	0.01	g N m^{-3}	Chen et al. 2014
K_{CH4}^{Db}	S_{CH4} affinity constant for DAMO bacteria	0.0384	g COD m^{-3}	Raghoebarsing et al. 2006
K_{O2}^{Db}	S_{O2} inhibition constant for DAMO bacteria	0.64	g COD m^{-3}	Lopes et al. 2011
$K_{I,NO2}^{Db}$	S_{NO2} inhibition constant for DAMO bacteria	57.4	g N m^{-3}	He et al. 2013
Anaerobic ammonium oxidizing bacteria (Anammox)				
μ_{An}	Maximum growth rate of Anammox	0.003	h^{-1}	Koch et al. 2000
b_{An}	Decay rate coefficient of Anammox	0.00013	h^{-1}	Hao et al. 2002
K_{NO2}^{An}	S_{NO2} affinity constant for Anammox	0.05	g N m^{-3}	Hao et al. 2002
K_{NH4}^{An}	S_{NH4} affinity constant for Anammox	0.07	g N m^{-3}	Strous et al. 1998
K_{O2}^{An}	S_{O2} inhibition constant for Anammox	0.01	g COD m^{-3}	Strous et al. 1998
$K_{I,NO2}^{An}$	S_{NO2} inhibition constant for Anammox	400	g N m^{-3}	Lotti et al. 2012
Methane oxidizing bacteria (MOB)				
μ_{MOB}	Maximum growth rate of MOB	0.0625	h^{-1}	Daelman et al. 2014
b_{MOB}	Decay rate coefficient of MOB	0.01	h^{-1}	Daelman et al. 2014
K_{CH4}^{MOB}	S_{CH4} affinity constant for MOB	0.24	g COD m^{-3}	Daelman et al. 2014
K_{O2}^{MOB}	S_{O2} affinity constant for MOB	0.2	g COD m^{-3}	Daelman et al. 2014
Sulfur oxidizing bacteria (SOB)				
μ_{SOB}^{R1}	Maximum reaction rate of R1	0.028	h^{-1}	Xu et al. 2013
μ_{SOB}^{R2}	Maximum reaction rate of R2	0.245	h^{-1}	Xu et al. 2014

μ_{SOB}^{R3}	Maximum reaction rate of R3	0.135	h^{-1}	Xu et al. 2014
μ_{SOB}^{R4}	Maximum reaction rate of R4	0.020	h^{-1}	Xu et al. 2014
μ_{SOB}^{R5}	Maximum reaction rate of R5	0.083	h^{-1}	Xu et al. 2014
b_{SOB}	Decay rate coefficient of SOB	0.002	h^{-1}	Wang et al. 2010
K_S^{R1}	S_S affinity constant for R1	11	g S m^{-3}	Xu et al. 2013
K_{O2}^{R1}	S_{O2} affinity constant for R1	200	g COD m^{-3}	Xu et al. 2013
K_S^{R2}	S_S affinity constant for R2	1.36	g S m^{-3}	Xu et al. 2014
$K_{I,S}^{R2}$	S_S inhibition constant for R2	2053.2	g S m^{-3}	Xu et al. 2014
K_{NO3}^{R2}	S_{NO3} affinity constant for R2	0.20	g N m^{-3}	Xu et al. 2014
$K_{I,NO2}^{R2}$	S_{NO2} inhibition constant for R2	0.698	g N m^{-3}	Xu et al. 2014
K_S^{R3}	S_S affinity constant for R3	1.80	g S m^{-3}	Xu et al. 2014
$K_{I,S}^{R3}$	S_S inhibition constant for R3	1.38	g S m^{-3}	Xu et al. 2014
K_{NO2}^{R3}	S_{NO2} affinity constant for R3	0.21	g N m^{-3}	Xu et al. 2014
$K_{I,NO2}^{R3}$	S_{NO2} inhibition constant for R3	0.65	g N m^{-3}	Xu et al. 2014
K_S^{R4}	X_S affinity constant for R4	0.215	g S m^{-3}	Xu et al. 2014
K_{NO3}^{R4}	S_{NO3} affinity constant for R4	0.183	g N m^{-3}	Xu et al. 2014
K_S^{R5}	X_S affinity constant for R5	174.51	g S m^{-3}	Xu et al. 2014
K_{NO2}^{R5}	S_{NO2} affinity constant for R5	0.107	g N m^{-3}	Xu et al. 2014

Table S5. System Performance of the MBfRs at Steady State under the Operational Conditions of Scenario 0 in Table 1

Characteristics/performance		Influent	Effluent
**Main-stream**	Ammonium, NH_4^+ (g N m^{-3})	50	3.6
	Methane, CH_4 (g COD m^{-3})	50	0.7
	Sulfide, S^{2-} (g S m^{-3})	30	0.3
	Nitrite, NO_2^- (g N m^{-3})	0	0
	Nitrate, NO_3^- (g N m^{-3})	0	0
	Sulfate, SO_4^{2-} (g S m^{-3})	0	29.7
	TN removal efficiency (%)	92.8	
	CH_4 removal efficiency (%)	98.6	
	S^{2-} removal efficiency (%)	99.0	
**Side-stream**	Ammonium, NH_4^+ (g N m^{-3})	1000	7.2
	Methane, CH_4 (g COD m^{-3})	100	0.2
	Sulfide, S^{2-} (g S m^{-3})	30	0.1
	Nitrite, NO_2^- (g N m^{-3})	0	6.1
	Nitrate, NO_3^- (g N m^{-3})	0	19.1
	Sulfate, SO_4^{2-} (g S m^{-3})	0	29.9
	TN removal efficiency (%)	96.8	
	CH_4 removal efficiency (%)	99.8	
	S^{2-} removal efficiency (%)	99.7	

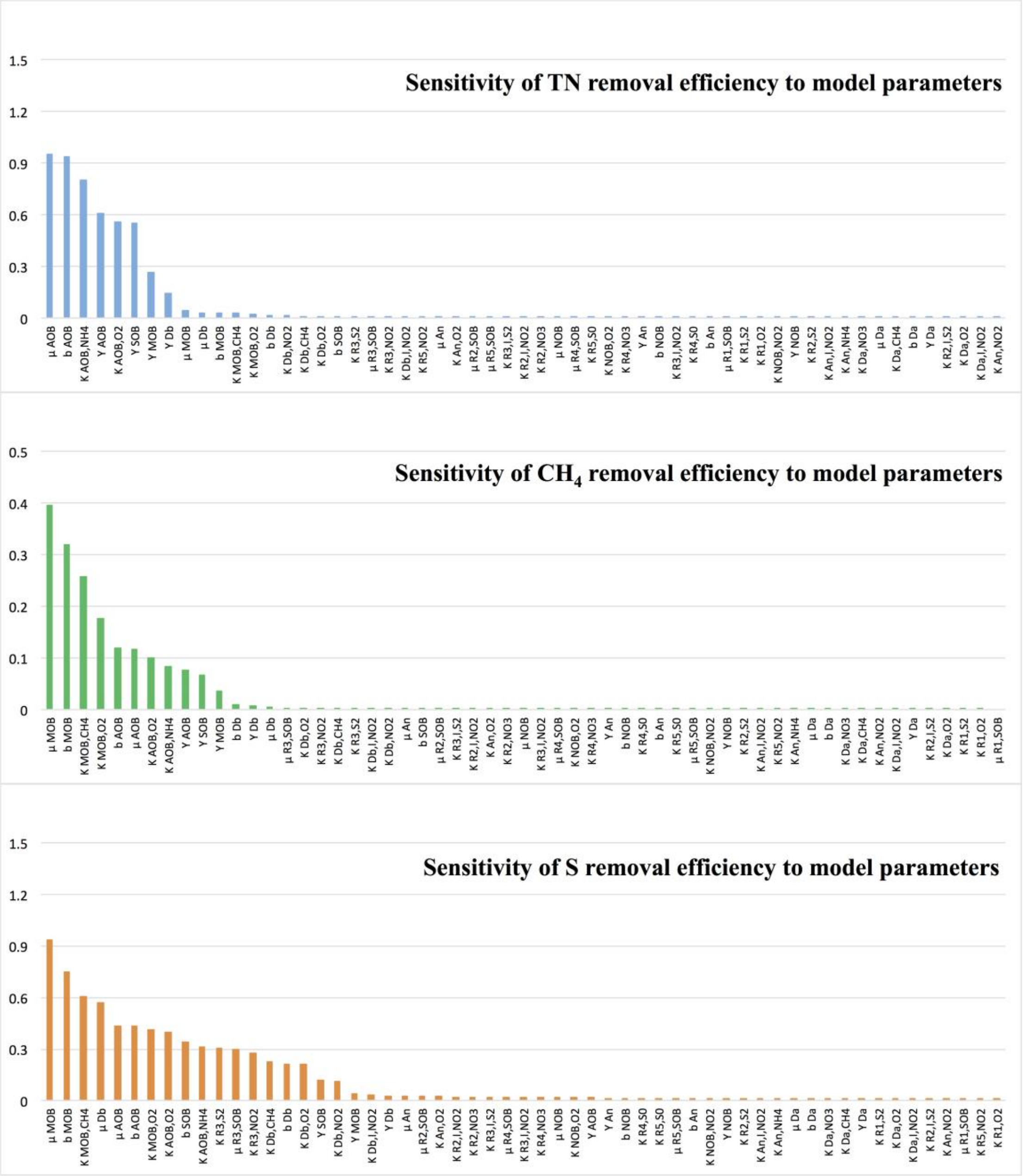

Figure S1. Sensitivity function for TN, dissolved methane, and sulfide removal efficiencies in the main-stream MBfR. The applied influent surface loading (L_{IN}), oxygen surface loading (L_{O2}), and biofilm thickness (L_f) are 0.0027 m d^{-1}, 0.52 g m^{-2} d^{-1}, and 300 µm, respectively.

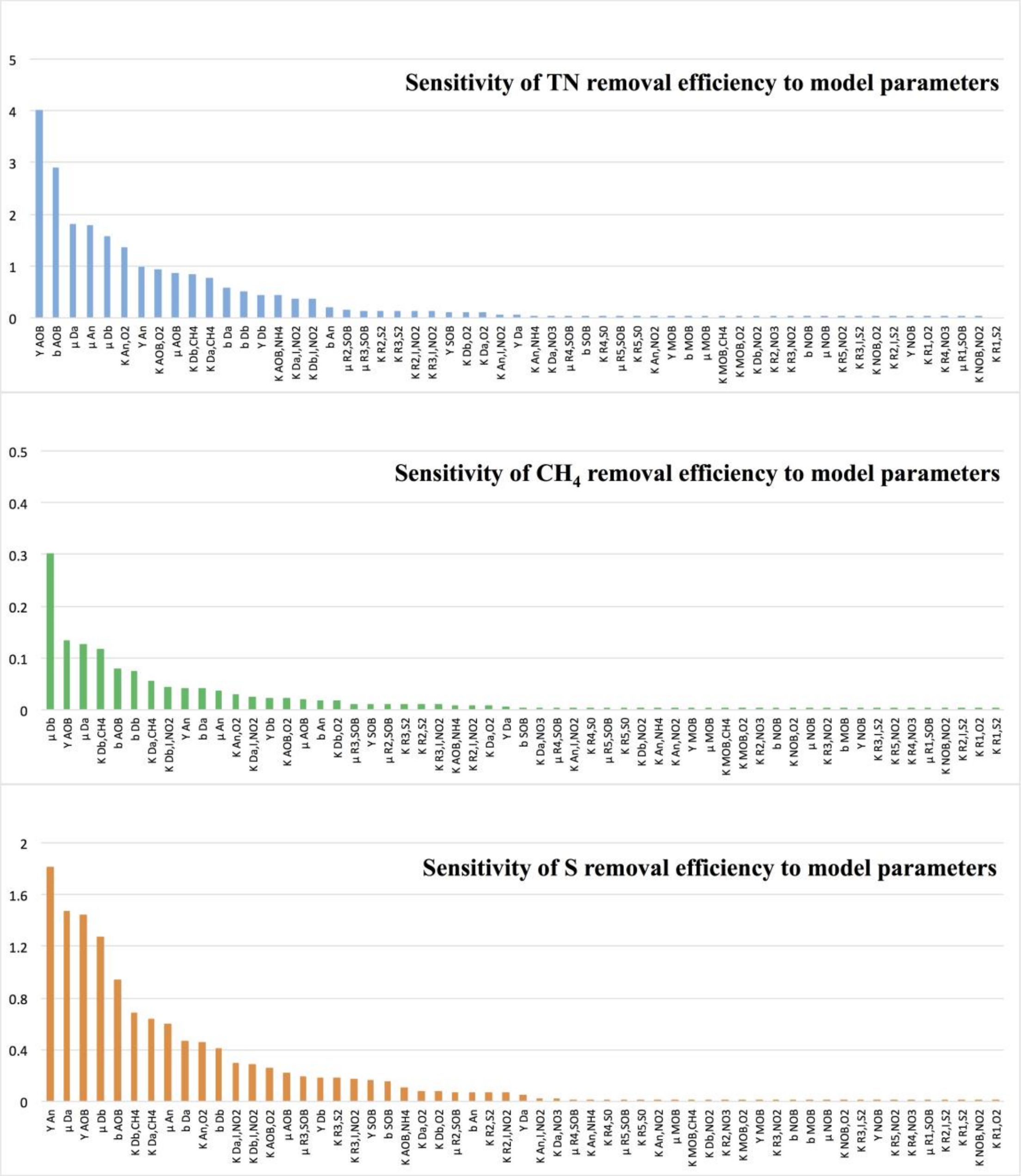

Figure S2. Sensitivity function for TN, dissolved methane, and sulfide removal efficiencies in the side-stream MBfR. The applied influent surface loading (L_{IN}), oxygen surface loading (L_{O2}), and biofilm thickness (L_f) are 0.001 m d^{-1}, 3.65 g m^{-2} d^{-1}, and 750 µm, respectively.

Additional References

Wiesmann, U. (1994) Biological nitrogen removal from wastewater. In Biotechnics/Wastewater, pp. 113-154, Springer Berlin Heidelberg.

Chen, X., Guo, J., Shi, Y., Hu, S., Yuan, Z. and Ni, B.-J. (2014) Modeling of Simultaneous Anaerobic Methane and Ammonium Oxidation in a Membrane Biofilm Reactor. Environmental Science & Technology 48(16), 9540-9547.

Strous, M., Heijnen, J.J., Kuenen, J.G. and Jetten, M.S.M. (1998) The sequencing batch reactor as a powerful tool for the study of slowly growing anaerobic ammonium-oxidizing microorganisms. Applied Microbiology and Biotechnology 50(5), 589-596.

Daelman, M.R.J., Van Eynde, T., van Loosdrecht, M.C.M. and Volcke, E.I.P. (2014) Effect of process design and operating parameters on aerobic methane oxidation in municipal WWTPs. Water Research 66(0), 308-319.

Xu, X.J., Chen, C., Lee, D.J., Wang, A.J., Guo, W.Q., Zhou, X., Guo, H.L., Yuan, Y., Ren, N.Q. and Chang, J.S. (2013) Sulfate-reduction, sulfide-oxidation and elemental sulfur bioreduction process: Modeling and experimental validation. Bioresource Technology 147, 202-211.

Wang, A.J., Liu, C.S., Ren, N.Q., Han, H.J. and Lee, D. (2010) Simultaneous removal of sulfide, nitrate and acetate: Kinetic modeling. Journal of Hazardous Materials 178(1-3), 35-41.

Henze, M., Gujer, W., Mino, T., van Loosdrecht, M.C.M., Henze, M., Gujer, W., Mino, T. and van Loosdrecht, M.C.M. (2000) Activated sludge models ASM1, ASM2, ASM2d and ASM3, IWA Publishing.

Raghoebarsing, A.A., Pol, A., van de Pas-Schoonen, K.T., Smolders, A.J.P., Ettwig, K.F., Rijpstra, W.I.C., Schouten, S., Damste, J.S.S., Op den Camp, H.J.M., Jetten, M.S.M. and Strous, M. (2006) A microbial consortium couples anaerobic methane oxidation to denitrification. Nature 440(7086), 918-921.

Lopes, F., Viollier, E., Thiam, A., Michard, G., Abril, G., Groleau, A., Prévot, F., Carrias, J.F., Albéric, P. and Jézéquel, D. (2011) Biogeochemical modelling of anaerobic vs. aerobic methane oxidation in a meromictic crater lake (Lake Pavin, France). Applied Geochemistry 26(12), 1919-1932.

He, Z., Cai, C., Geng, S., Lou, L., Xu, X., Zheng, P., Hu, B. (2013) Modeling a nitrite dependent anaerobic methane oxidation process: Parameters identification and model evaluation. Bioresource Technology 147, 315-320.

Koch, G., Egli, K., Van der Meer, J.R. and Siegrist, H. (2000) Mathematical modeling of autotrophic denitrification in a nitrifying biofilm of a rotating biological contactor. Water Science and Technology 41(4-5), 191-198.

Hao, X., Heijnen, J.J. and van Loosdrecht, M.C.M. (2002) Sensitivity analysis of a biofilm model describing a one-stage completely autotrophic nitrogen removal (CANON) process. Biotechnology and Bioengineering 77(3), 266-277.

Lotti, T., van der Star, W.R.L., Kleerebezem, R., Lubello, C., van Loosdrecht, M.C.M. (2012) The effect of nitrite inhibition on the anammox process. Water Research 46, 2559-2569.

Xu, X.J., Chen, C.A., Wang, A.J., Guo, W.Q., Zhou, X., Lee, D.J., Ren, N.Q. and Chang, J.S. (2014) Simultaneous removal of sulfide, nitrate and acetate under denitrifying sulfide removal condition: Modeling and experimental validation. Journal of Hazardous Materials 264, 16-24.
